# Case report: mixed large-cell neuroendocrine and hepatocellular carcinoma of the liver

**DOI:** 10.3389/fonc.2023.1309798

**Published:** 2024-01-09

**Authors:** Xin Gao, Heng Wang, Zheyu Niu, Meng Liu, Xiaohan Kong, Hongrui Sun, Chaoqun Ma, Huaqiang Zhu, Jun Lu, Xu Zhou

**Affiliations:** ^1^ Department of Hepatobiliary Surgery, Shandong Provincial Hospital, Shandong University, Jinan, China; ^2^ Department of Hepatobiliary Surgery, Shandong Provincial Hospital, Shandong First Medical University, Jinan, China; ^3^ Department of Clinical Research, Qilu Synva Pharmaceutical Co. Ltd., Dezhou, China; ^4^ Department of Physiology and Pathophysiology, School of Basic Medical Sciences, Cheeloo College of Medicine, Shandong University, Jinan, Shandong, China

**Keywords:** hepatocellular carcinoma, neuroendocrine carcinoma, genetic testing, chemotherapy, rare disease

## Abstract

**Background:**

Cases of large-cell neuroendocrine carcinoma (NEC) concomitant hepatocellular carcinoma (HCC) are very rare. Based on the microscopic characteristics, mixed HCC-NEC tumors can be divided into collision type and combined type. We report a patient with both collision and combined type HCC-NEC tumor at the same time.

**Case presentation:**

A 58-year-old man with hepatitis B and cirrhosis was found to have two masses in segment 5 and segment 8 of the liver, respectively. Preoperative imaging diagnosis was primary liver cancer. Indocyanine green retention test (ICG R_15)_ <10% suggested that the patient can tolerate surgery. Partial hepatectomy was performed under the guidance of 3D reconstruction. Postoperative pathology showed that most of the tumors in S5 were large-cell neuroendocrine carcinoma (90%), and a small part were hepatocellular carcinoma (10%). The tumor in S8 of the liver was diagnosed as HCC combined with immunohistochemistry. After surgery, the patient underwent genetic testing, which indicated mutations in TP53 gene. The test of immune markers of the sample suggest that the patient may benefit little from immune checkpoint inhibitor therapy. The cisplatin and etoposide chemotherapy protocol to the patient following their surgery. Eight month later after the operation, Enhanced CT showed there was no recurrence or metastasis of the tumor.

**Conclusion:**

The case at hand augments the understanding of HCC-NEC mixed tumors, offering pivotal insights into their precise diagnosis and treatment modalities. Furthermore, we document a favorable prognosis, marked by an absence of recurrence signs thus far—a rarity in comparable instances. This enlightenment stands to facilitate the handling of ensuing cases and enhance patient prognoses.

## Introduction

Hepatocellular carcinoma stands as the predominant malignant liver tumor, whereas the occurrence of intrahepatic cholangiocarcinoma is also noted with some regularity. Nevertheless, other variants of liver malignancies remain notably scarce. The concomitance of HCC and NEC is exceptionally uncommon, evidenced by a mere 30 instances reported hitherto. Conventionally, HCC is identified pre-resection and is associated with a disadvantageous prognosis (1). HCC-NEC neoplasms are categorized as either combined or collision types. The former encompasses 2.0-3.6% of primary liver malignancies, while the latter, constituting type 2 tumors, manifests at a remarkably minimal incidence rate of 0.1-1% (2, 3). Here, we present a case of HCC-NEC, provide a detailed description, and include genetic testing to offer effective guidance for improving patient prognosis. Additionally, we reviewed previous reports of related cases.

## Case description

**Figure 1:**
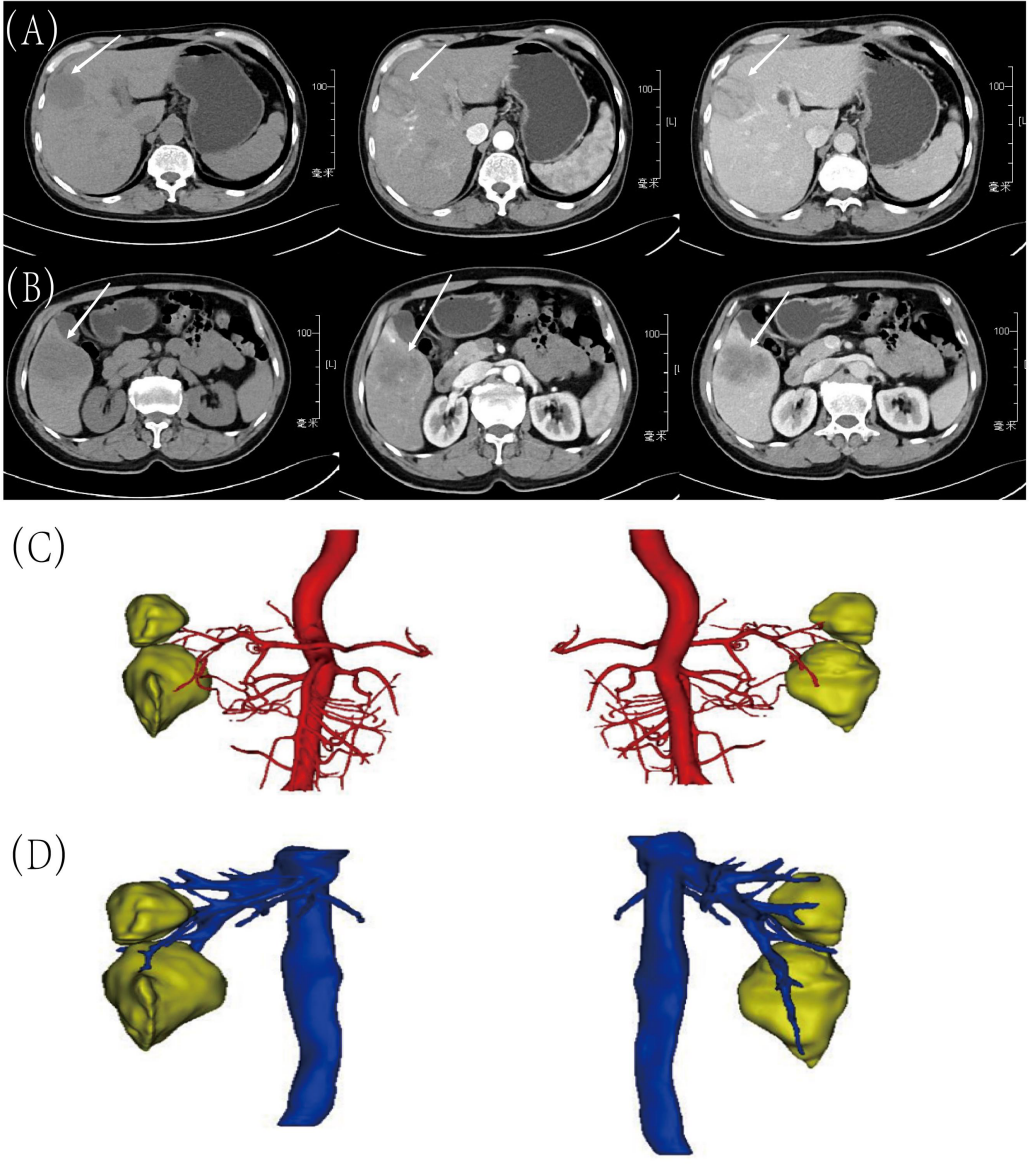
Preoperative CT images and Three-Dimensional Reconstructed CT. **(A)** Tumor in S8: before contrast, arterial phase and portal venous phase; **(B)** Tumor in S5: before contrast, arterial phase and portal venous phase; **(C)** Arterial relationships with tumors on 3D Reconstructed Images; **(D)** The relationship between veins and tumors on 3D Reconstructed Images.

A 58-year-old man presented to our hospital with right upper abdominal pain for two months and two hepatic masses that had been detected by liver computed tomography (CT) on the S5 and S8 segments. Three senior radiologists conducted an evaluation of the lesions based on the CT/MRI LI-RADS (Version 2018) criteria. All three doctors concurred that both lesions were classified as LR-4. The patient had a 30-year history of chronic hepatitis B without treatment and regular check-ups. The patient denied a family history of cancer. The serum level of alpha-fetoprotein (AFP), CA19-9, CA125, and CEA were normal. However, PIVKA-II was slightly elevated (516 mAU/ml). The patient had normal liver enzymes, total serum bilirubin of 15.1 umol/l, and serum albumin of 41.3 umol/l. The preoperative liver function was graded as Child-Pugh A. The preoperative liver computed tomography (CT) shows that there were two masses in the liver and the imaging diagnosis was liver cancer. In the junction of the left and right lobes of the liver and in the lower segment of the right anterior lobe of the liver, patchy and slightly low-density lesions with unclear boundaries were observed, with sizes of about 4.1×3.6cm and 5.7×3.7cm, respectively. The image of tumor in S8 was shown in **Figure 1A**. In the latter lesions, the enhancement in the arterial phase showed obvious uneven enhancement, and the enhancement in the venous phase and the delayed phase decreased, showing relatively low density (**Figure 1B**). CT of the chest and extrahepatic abdomen showed no other lesions to rule out metastatic tumors. Three-Dimensional Reconstructed CT of liver parenchyma, tumor, and vessels showed that there was no variation in hepatic artery, hepatic vein, and portal vein (**Figures 1C, D**). The volume of the liver totaled 1513.6ml. We simulated anatomic hepatectomy through 3D reconstruction, requiring removal of the S4, S5, S6, and S8 of the liver, for a total of 991.4ml. The patient has cirrhosis and anatomic hepatectomy may result in insufficient residual liver ratio.

The patient exhibited good liver function preoperatively (Child-Pugh Grade A) and no evidence of lymph node or vascular metastasis was found, making surgical resection feasible. Given the patient’s cirrhosis and the results of the 3D reconstruction, anatomical segmentectomy was deemed risky due to insufficient residual liver function, posing a risk of postoperative liver failure. Therefore, we decided to perform a partial hepatectomy. Laparoscopic examination revealed cirrhosis without ascites. The tumors were located in segments S5 and S8 of the liver, with the S5 tumor being particularly large. After attempting laparoscopic surgery, we switched to open surgery due to difficulties in exposure, potential for significant bleeding, and the challenge of ensuring therapeutic efficacy laparoscopically. The liver parenchyma was excised using an ultrasonic knife approximately 2cm from the tumor margin, ensuring complete tumor removal.

**Figure 2:**
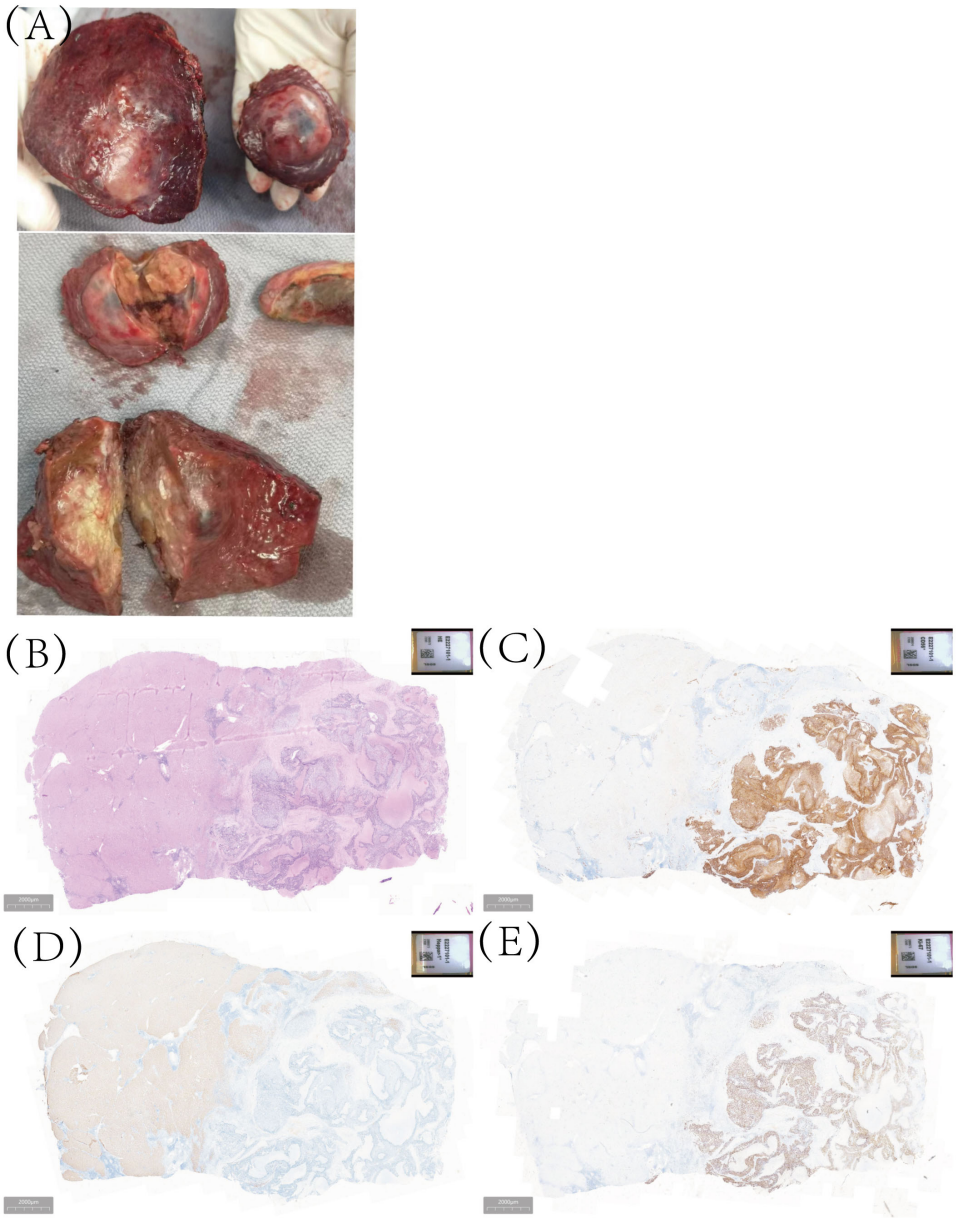
The gross examination of the surgical specimen, as well as histopathological analysis and immunohistochemical examination, the tumor in S5 comprises two distinct components: large-cell neuroendocrine carcinoma and hepatocellular carcinoma. **(A)** The macroscopic specimen; **(B)** Hematoxylin-eosin staining; **(C)** for CD56 staining; **(D)** Heppar-1 staining; **(E)** Ki-67 staining.

To the naked eye, the tumor in S5 and S8 were solid and yellowish-white in color with some necrotic changes (**Figure 2A**). Postoperative pathology showed that the tumor in S5 was 7×3.5cm, with poor differentiation and large necrosis. Large cell neuroendocrine carcinoma was found in most areas (90%) and hepatocellular carcinoma in a small part (10%). Immunohistochemistry shows CgA (-),CD56 (+diffuse), Ki-67 (+80%), Heppar-1 (+) (**Figures 2B–E**). There was no fibrous tissue separation at the tumor junction. Combined with immunohistochemistry, it was consistent with mixed hepato-neuroendocrine tumor (MiNET). The tumor in S5 was 4.5×4.5cm with Heppar-1(+), Hepatocyte (+), and Ki67 (+8%). Final diagnosis was hepatocellular carcinoma, grade 2.

## Targeted drug detection

**Figure 3:**
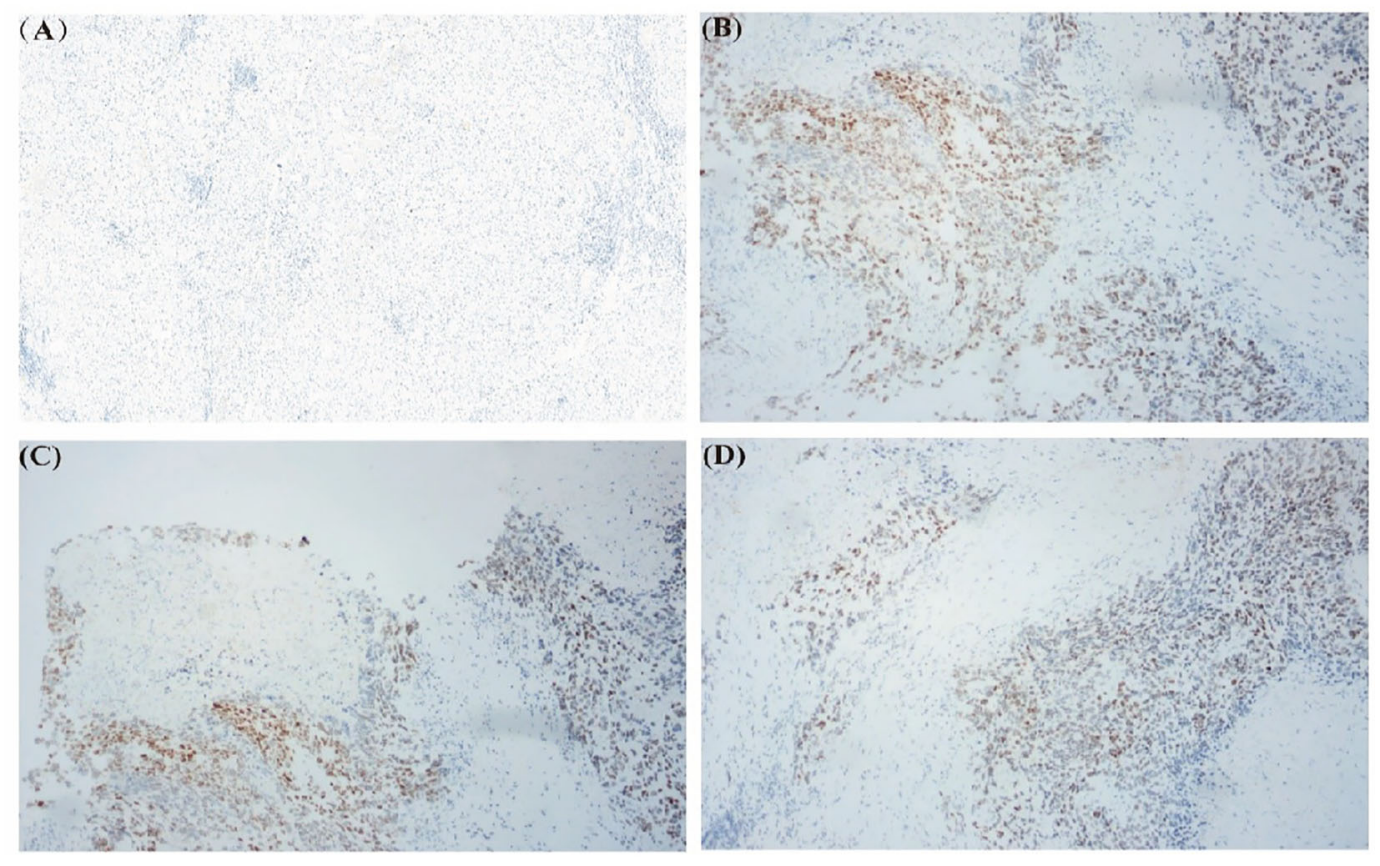
Immunohistochemical staining of TP53 gene in excised specimens. **(A)** TP53 gene staining in paracancer sample. **(B-D)** TP53 gene staining in tumor sample.

Due to the rarity of HCC-NEC and the lack of recognized systematic treatment options other than surgical treatment, genetic testing is recommended for patients to guide subsequent treatment. DNA samples were isolated from the peripheral blood cells and excision specimen of the patient. The exon region, partial intron region and fusion breakpoint region of 1059 genes related to tumorigenesis and development were comprehensively detected. Sequence analysis revealed missense mutation of TP53 gene (p.I232F, mutation abundance:34.32%). Preclinical studies or case reports have shown that patients with TP53 gene mutation are sensitive to the therapy of AZD1775+ carboplatin, unfortunately, as of now, AZD1775 remains unapproved for use in the market, thus limiting our ability to administer this drug to patients. Immunohistochemical staining of TP53 gene was performed on both cancerous and paracancer samples. We found that p53 gene was almost not expressed in paracancer sample, while it was expressed in tumor sample (**Figure 3**).

## Detection of immune markers

This sample had a low Tumor Mutation Burden of 5.1 muts/Mb and the Microsatellite Instability (MSI) status was MSS/MSI-L, The detection results of PD-1 protein expression showed that TPS was < 1%, CPS was 3. The test of immune markers of the sample suggest that it may benefit little from immune checkpoint inhibitor therapy.

Regrettably, despite utilizing various diagnostic measures including genetic testing and immunohistochemical staining, we were unable to establish an accurate and efficacious treatment plan for the patient. Following consultation of the latest guidelines for neuroendocrine tumor treatment, we ultimately decided to administer a combination of cisplatin and etoposide chemotherapy.

**Figure 4:**
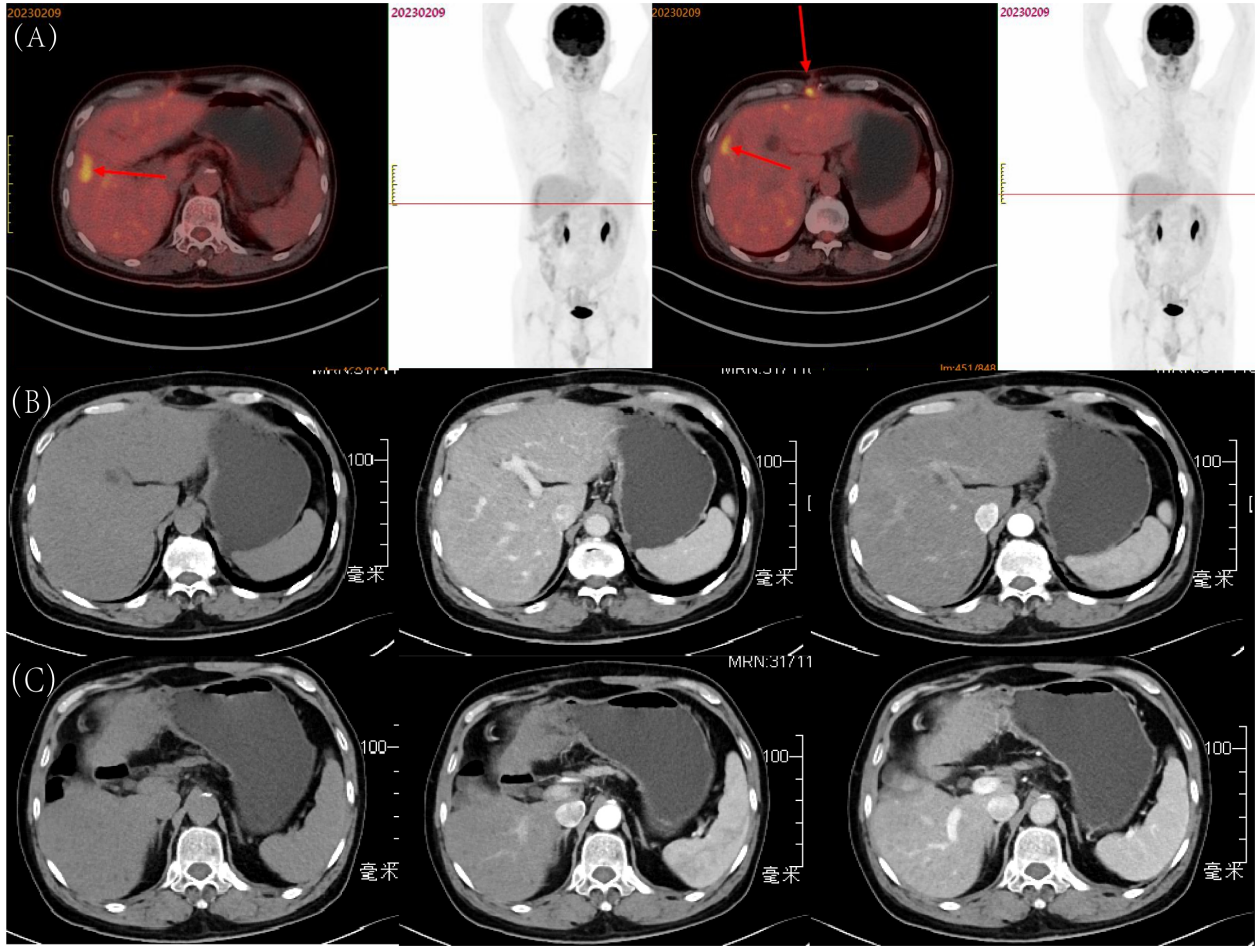
Postoperative Follow-up of the Patient: **(A)** Whole-body PET-CT examination three months after surgery; **(B)** Tumor in S8, eight months after surgery: before contrast, arterial phase, and portal venous phase; **(C)** Tumor in S5, eight months after surgery: before contrast, arterial phase, and portal venous phase.

Three months after the patient’s surgery, we performed a whole-body PET-CT examination on the patient (**Figure 4A**). The final results showed that the FDG metabolism in the surgical area was high, and the corresponding parts appeared as low-density areas on CT, and the FDG metabolism in the abdominal wall muscles in the surgical area was increased. It was considered in response to the postoperative reaction, no tumor manifestations were found in other areas, which further confirmed that the neuroendocrine tumor at the liver resection site in this case was primary to the liver. To date, eight months post-surgery, the patient has not exhibited any significant signs of recurrence (**Figures 4B, C**).

## Discussion

Primary neuroendocrine tumors situated in the liver are exceedingly scarce, representing a mere 0.4% of primary lesions in liver resections (4). Prior to confirming a diagnosis of HCC-NEC tumors, it is imperative to first exclude the possibility of metastatic NEC through the utilization of PET-CT or whole-body enhanced CT (5). Given the negligible differences in preoperative CT imaging findings between NEC and HCC, establishing an accurate diagnosis pre-surgery proves challenging, predominantly relying on postoperative pathological assessments (6).

HCC-NEC tumors have historically been bifurcated into combined and collision types, contingent on their histopathological characteristics. A combined HCC-NEC is characterized by the concurrent presence of HCC and NEC elements in direct juxtaposition, often featuring a transitional zone depicting the amalgamation of the two cellular types. Conversely, collision HCC-NEC tumors manifest a complete demarcation between HCC and NEC components by fibrous septa, devoid of any transitional zone (4). A tertiary pathological variant, termed the ‘intermediate type,’ is documented, marked by the co-expression of HCC and NEC markers in undifferentiated cancerous cells. In the pathological tissues we studied, the two tumor cell variants were intermixed and thus, categorized as mixed HCC-NEC tumors. The genesis of such tumors remains ambiguous, with two prevailing conjectures presented in scholarly works (5). One postulation posits that intrahepatic neuroendocrine tumors emanate from the dedifferentiation of hepatic bile duct epithelial cells. Alternatively, a hypothesis suggests their inception through the metamorphosis of other malignant hepatic tumor variants. The case under our scrutiny exhibited two discrete sites on the liver, with one primarily comprising hepatocellular carcinoma, and the other predominantly featuring neuroendocrine tumor elements. In light of the immunohistochemistry findings, our case aligns with the latter hypothesis, implying that the neuroendocrine tumor elements are consequential to the transformation of hepatocellular carcinoma components.

We meticulously reviewed a total of 30 publications pertinent to HCC-NEC tumors, accessible in databases including PubMed and Web of Science, with the coverage extending from 1984 to 2023 (refer to **Table 1**). A thorough analysis was conducted, examining the fundamental and pathological attributes, treatment modalities, and prognoses of the depicted cases. A striking observation is the conspicuous scarcity of female patients, accounting for merely 9.6%, suggesting a potential linkage between the tumor occurrence and specific male characteristics, warranting additional exploratory studies. Moreover, a strong correlation is discerned between the incidence of tumors and chronic hepatitis infection, marked at 70.9%.

**Table 1:** Clinicopathological analysis of 31 patients with HCC-NEC.

Reference, year	Age	Sex	Chronic hepatitis	Diagnosis	Tumor size (cm)	Type	HCC differentiation
Barsky et al.,1984 (4)	43	M	HBV	autopsy	Large	combined	NA
Artopoulos et al.,1994 (5)	69	M	HBV	FNA	10	combined	NA
Vora et al.,2000 (6)	63	M	NA	NA	10	combined	NA
lshida et al., 2003 (7)	72	M	HCV	Resection	3	collision	mod
Yamaguchi et al., 2004 (8)	71	M	HCV	Resection	4.1	combined	mod
Garcia et al., 2006 (9)	50	M	HCV	Resection	5.3	collision	mod
Yang et al., 2009 (10)	65	M	HBV	Resection	7.5	combined	mod
Tazi et al., 2011 (11)	68	M	HBV	Resection	4	collision	NA
Nakanishi et al., 2012 (12)	76	M	HCV	Resection	3	combined	mod
Hammedi et al., 2012 (13)	51	M	HCV	Resection	20	combined	NA
Aboelenen et al., 2014 (14)	56	F	N	biopsy	NA	combined	mod
Yun et al., 2016 (15)	68	F	HBV	Resection	2.5	combined	NA
Nishino et al., 2016 (16)	72	M	HCV	Resection	2.5	combined	NA
choi et al., 2016 (17)	72	M	HCV	Resection	2.5	collision	mod
Nomura et al., 2016 (18)	71	M	HCV	Resection	4	combined	mod-por
	71	M	HCV	Resection	3	collision	por
	58	M	HBV	Resection	4.3	combined	NA
	50	M	HBV	Resection	1.8	combined	mod-por
	63	M	HCV	Resection	3	combined	por
Baker et al., 2016 (19)	76	M	N	Resection	5.5	collision	mod
Liu et al., 2016 (1)	65	M	HCV	Resection	4.3	collision	well-por
Beard et al., 2017 (20)	19	M	N	Resection	25	combined	NA
Okumura et al., 2017 (21)	70	M	HCV	Resection	11	coll&com	mod-por
Lu et al., 2017 (22)	65	M	N	biopsy	14	combined	por
Yilmaz et al., 2018 (23)	56	M	N	Resection	2.3	collision	mod
Kwon et al., 2018 (24)	44	M	HBV	Resection	10.5	combined	por
Jahan et al., 2020 (25)	50	M	HCV	Resection	2.7	combined	mod
Nakano et al.,2021 (26)	84	F	N	Resection	5.5	combined	mod
Ikeda et al.,2021 (27)	79	M	N	Resection	6	combined	NA
Shin et al.,2023 (28)	63	M	N	Resection	9	combined	por
Present case	58	M	HBV	Resection	5.7	combined	mod

M, male; F, female; LN, lymph node; NA, not applicable; FNA, fine needle aspiration.

It was discerned that all the instances of HCC-NEC were preliminarily categorized as straightforward HCC based on preoperative imaging, with the refined diagnosis of HCC-NEC being attained solely via needle biopsy or subsequent pathological evaluations. This phenomenon intimates the probability of a more extensive pool of undiagnosed patients, potentially suffering from this variant of HCC, receiving treatment for straightforward HCC. This revelation underscores the imperative for enhanced research into the imaging manifestations of this ailment and HCC, fostering early detection of HCC-NEC and subsequently expanding therapeutic avenues for patients. Research conducted by Nakano et al. has illuminated that HCC-NEC does portray distinctive features in preoperative imaging, suggesting a feasible adaptation of imaging diagnostic criteria for more precise disease identification. Additionally, we observed that the AFP levels in our patient constantly stayed within the normal range, fluctuating between 1.2 and 1.3 ng/ml. Importantly, the patient’s preoperative PIVKA-II level was elevated to 516 mAU/ml, which was the only tumor marker detected to be elevated before surgery. This value dropped to normal (28 mAU/ml) in a check-up two months post-surgery. Regrettably, other studies have not detailed the status of these two tumor markers. We hope that future, more comprehensive reports on HCC-NEC will provide deeper insights, further revealing the relationship between the progression of HCC-NEC and tumor markers.

In terms of adjuvant therapy for HCC-NEC after surgery, there is still a lack of definitive conclusions. Considering its unique histological characteristics, HCC-NEC may not respond well to traditional treatments for hepatocellular carcinoma. Therefore, taking into account the neuroendocrine component of the cancer, we chose a combination therapy of cisplatin and etoposide. This combined treatment, utilizing the efficacy of both drugs, could be more effective against the complex nature of HCC-NEC, which includes neuroendocrine elements. Furthermore, given the rarity of HCC-NEC and its potential resistance to conventional treatments, we believe that using cisplatin plus etoposide as an adjuvant chemotherapy regimen may offer a more comprehensive treatment option for patients, potentially improving prognosis. Although our case showed positive outcomes in the short term, we recognize the need for longer follow-up and further studies involving more cases to validate the long-term effectiveness and applicability of this treatment approach.

Out of the 31 cases analyzed, follow-ups were conducted for 27 patients. Within this subset, 14 exhibited evident signs of recurrence, 10 succumbed within the first postoperative year, and a mere three surpassed the three-year survival threshold (detailed in **Table 2**). Given the constrained sample dimension and the predominantly short-term follow-up duration, our approximation of the one-year postoperative survival rate is limited to 40.7% (omitting four patients with indeterminate prognoses). It is imperative to recognize that, as per diverse studies, the three-year survival metric for hepatic cell carcinoma patients remains approximately at 50.8% (4), signaling a substantially diminished prognosis for neuroendocrine tumor patients. While our patient demonstrated no recurrence indicators through an eight-month follow-up interval, the prospect of the follow-up duration being insufficient cannot be overlooked. As such, sustained observation of the patient’s condition is underway.

**Table 2:** Clinical treatment and prognosis of 31 patients with HCC-NEC.

Reference, year	Matasta-sis	Treatment	Recurrence site	Survival	OS (mon)
Barsky et al.,1984 (4)	Omentum	Palliative chemotherapy d/t omental seeding		Dead	26
Artopoulos et al.,1994 (5)		Resection	NA	NA	NA
Vora et al.,2000 (6)	NA	Resection	NA	Dead	1
lshida et al., 2003 (7)		Resection	NA	NA	NA
Yamaguchi et al., 2004 (8)	LNs	Resection	bone,5 mon	Alive	5
Garcia et al., 2006 (9)		Resection	liver,peritoneum 4 mon	Alive	16
Yang et al., 2009 (10)	LNs	Resection	liver,adrenal,LN 4 mon	Dead	12
Tazi et al., 2011 (11)	LNs	Resection	No recurrence	Alive	28
Nakanishi et al., 2012 (12)		TACE followed by resection	bone,7 mon	Dead	17
Hammedi et al., 2012 (13)		Resection	NA	Dead	1
Aboelenen et al., 2014 (14)		Resection	No recurrence	Alive	6
Yun et al., 2016 (15)		Resection	bone,6 mon	Dead	2
Nishino et al., 2016 (16)	LNs	Resection	LN,0.7 mon	Dead	13
choi et al., 2016 (17)		Resection	liver,6 mon	Alive	10
Nomura et al., 2016 (18)	NA	Resection	liver,NA	Dead	10
	NA	RFA followed by resection	liver,NA	Dead	8.6
	NA	Resection	No recurrence	Alive	19.4
	NA	Resection	No recurrence	Alive	19.3
	NA	Resection	No recurrence	Alive	23.7
Baker et al., 2016 (19)		Resection	NA	NA	NA
Liu et al., 2016 (1)	LNs	Resection	Hepatic and renal failure	Dead	1.3
Beard et al., 2017 (20)	LNs	Resection	LN,4 mon	Alive	8
Okumura et al., 2017 (21)		Resection	bone,1 mon	Dead	3
Lu et al., 2017 (22)	gallbladder, portal vein	Hospice	NA	NA	NA
Yilmaz et al., 2018 (23)		Liver transplant	No recurrence	Alive	10
Kwon et al., 2018 (24)		Resection	liver and bone,1.9 mon	Dead	4
Jahan et al., 2020 (25)		Resection	liver, LN and bone,13 mon	Dead	33
Nakano et al.,2021 (26)		Resection	No recurrence	Alive	9
Ikeda et al.,2021 (27)		Resection	NA,4 mon	Dead	4
Shin et al.,2023 (28)		Resection	adrenal, liver and lung,5 mon	Dead	12
Present case		Resection	No recurrence	Alive	9

LN, lymph node; NA, not applicable; OS, overall survival; RFA, radiofrequency ablation; TACE, hepatic arterial chemoembolization.

In conclusion, instances of HCC-NEC remain a rarity, with a manifest gap in the scientific exploration concerning diagnosis, survival metrics, treatment modalities, among others. The anticipation persists that advancements in diagnostic methodologies will facilitate earlier identification of such cases, and a multifaceted approach, encompassing interventional embolization, surgical interventions, and pharmacotherapy, will potentially enhance patient survival rates.

## Data availability statement

The original contributions presented in the study are included in the article/supplementary material. Further inquiries can be directed to the corresponding author.

## Ethics statement

Written informed consent was obtained from the individual(s) for the publication of any potentially identifiable images or data included in this article.

## Author contributions

XG: Writing – original draft. HW: Writing – original draft. ZN: Validation, Writing – review & editing. ML: Data curation, Writing – review & editing. XK: Data curation, Writing – review & editing. HS: Data curation, Writing – review & editing. CM: Supervision, Writing – review & editing. HZ: Supervision, Writing – review & editing. JL: Supervision, Writing – review & editing. XZ: Supervision, Writing – review & editing.
